# Effect of Yak Skin Gelatin with Different Molecular Weights on the Properties of Gelatin/Polymethyl Vinyl Ether-*alt*-maleic-anhydride Copolymer Composite Scaffold Material

**DOI:** 10.1021/acsomega.4c06149

**Published:** 2025-03-03

**Authors:** Yuxia Zhang, Songhao Liu, Lin Rong, Liang Gao, Lixin Wei, Yuzhi Du, Hongxia Yang

**Affiliations:** a Northwest Institute of Plateau Biology, Chinese Academy of Sciences, Xining 810008, China; b University of Chinese Academic of Sciences, Beijing 100049, China; c School of Energy and Electrical Engineering, Qinghai University, Xining, Qinghai 810016, China

## Abstract

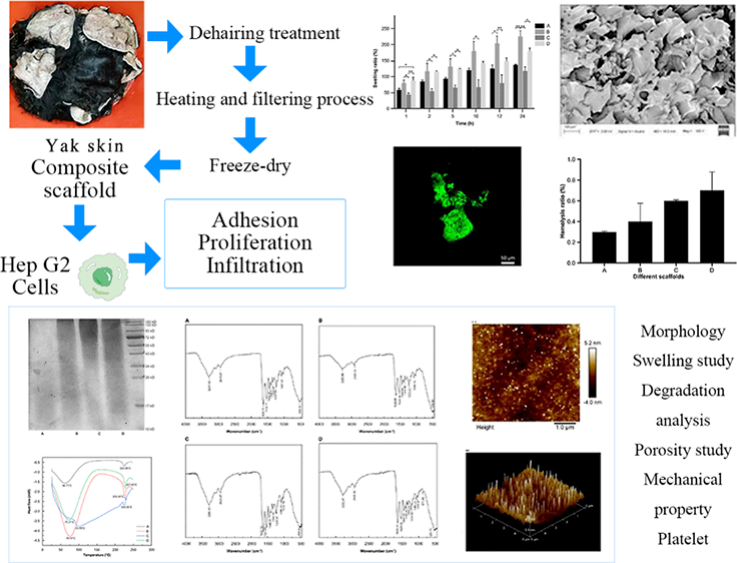

Gelatin has been extensively documented for its utility in tissue engineering applications. However, the exploration of yak skin gelatin as a novel gelatin source remains under-reported, particularly regarding the impact of varying molecular weights on the attributes of composite scaffold materials. This study investigates the distinctive behaviors of yak skin gelatin fractions with different molecular weights, assessing fundamental properties through electrophoretic analysis, thermodynamic property assessment, amino acid profiling, infrared spectroscopy, and atomic force microscopy. Then, the polymethyl vinyl ether-*alt*-maleic-anhydride copolymer (PMVE-MA) was introduced to fabricate the composite scaffold materials. It was observed that the hemolysis rate escalated with increasing gelatin molecular weight. Additionally, properties such as platelet adhesion and mechanical stability exhibited a molecular-weight-dependent threshold behavior. Importantly, no cytotoxic effects were observed across all groups. Notably, scaffold materials fabricated by gelatin with a molecular weight range of 0.1–0.22 μm demonstrated superior mechanical strength and cell adhesion, positioning them as optimal candidates for biodegradable vascular scaffold applications.

## 1 Introduction

Cardiovascular disease is a type of disease that affects the circulatory system, targeting the heart and blood vessels. The disease has one of the highest mortality rates in the world.^1^ It is estimated that there are ∼330 million people suffering from cardiovascular disease in China alone, and the disease follows an upward phase^2^. Typically, heart-related diseases, such as cardiovascular disease, cause lesions to develop on the heart, blood vessels, and other critical components of the circulatory system. Because the circulatory system connects through the entire human body, these lesions can occur many different places, resulting in a diverse range of symptoms in response to variations of the disease^3^. Consequently, treatment methods differ greatly among different variations and stages of the disease and are extremely complex; different treatments sometimes have serious impacts on the health and quality of life of patients, particularly middle-aged and elderly people.^4,5^

Treatments for cardiovascular disease include various courses of drug therapy, as well as surgical options. Surgical treatment generally includes percutaneous balloon coronary angioplasty, coronary stent implantation, and rotational atherectomy^3^. These surgical options are the most common source of blood supply treatment for patients with coronary atherosclerotic heart disease. A coronary stent implantation is commonly conducted for treating heart disease caused by myocardial ischemia, hypoxia, or necrosis due to vascular stenosis or obstruction, where reconstruction is required. Stent implantation is one of the preferred clinical treatments for arteriosclerosis among other diseases. Implantation is done where stenosis has occurred in the artery to expand and support the blood vessel, thereby unblocking the vessel and allowing blood to flow freely^5^. Stents have been used to treat a range of diseases in the circulatory system, including infarction, myocardial ischemia, and heart failure caused by an insufficient blood supply. Existing types of vascular stents used are primarily manufactured from biomedical metal tissue engineering, synthetic polymer material, and natural polymer material^6^. Biomedical metal tissue engineering stents were the earliest designs for vascular stents and are still widely used in clinical practice. Postoperative recovery is generally quick, with the benefit that the stent can be used as a drug carrier^7^. However, the biomedical metal tissue material of the stent is limited in terms of its refractory nature, development of postoperative restenosis, and late embolization. In response, recent years have seen the emergence of degradable metal stents.^8,9^ A fully absorbable biological stent was recently approved by the U.S. Food and Drug Administration (FDA) and is believed to lead the fourth revolution in coronary intervention^10^. As a natural biological material, gelatin has the advantages of natural composition, good biocompatibility, and a natural degradation process in the environment. It is a smart option for tissue engineering in general but particularly for blood vessel scaffolds.

Yunoki et al. (2003) and Nagai et al. (2008 and 2009) conducted research on the extraction of salmon skin collagen and found that the use of cross-linking agents during the formation of collagen fibers can raise the denaturation temperature of salmon skin collagen from 19 to 47 °C.^11−14^ Further, the studies revealed that with improved thermal stability, salmon skin collagen can be used to prepare collagen vascular grafts.^12−14^ Continued research on cytocompatibility and animal implantation has shown that salmon skin collagen can be used as a natural biomaterial in vascular tissue engineering and specifically for preparing vascular stents. Gelatin is found widely in nature and its species, across both terrestrial and marine environments^15^. One such species is the Yak, a type of cattle unique to the Qinghai–Tibet Plateau in China. Yak gelatin is produced in its skin through a series of internal processes and is rich in human essential amino acids in addition to various trace elements^16^. This specific type of gelatin serves a critical recovery function within the immune and hematopoietic system of the human body.^17,18^ Compared with other mammals, gelatin extracted from fresh yak skin is rich in amino acids and consequently has a higher thermal stability level^19^. Our research teams studied obtained yak skin gelatin under different enzymatic hydrolysis time periods and found that the denaturation temperature of skin gelatin is 66.38–120.52 °C. This temperature range is much higher than the denaturation temperature of fish skin gelatin or collagen, meaning that yak skin gelatin itself has a high denaturation temperature.

The polymethyl vinyl ether-*alt*-maleic-anhydride copolymer (PMVE-MA) is a type of FDA-approved acid glycoside polymer that is biodegradable and has low toxicity and high biocompatibility. It helps pure gelatin overcome the shortcomings, including being soluble in water and lacking adequate mechanical strength, making it suitable for vascular stent applications. Additionally, PMVE-MA also holds the capacity to improve drug adhesion and to act as a drug carrier so that it has been applied in studies for healing wounds in humans^20^ and has been used to encapsulate drugs to enhance bioadhesion^21^, indicating its utility in biomedical and drug delivery applications.

During the initial phase of our study, a subset of our team focused on examining the physicochemical properties of yak skin gelatin with varying molecular weights by manipulating the duration of enzymatic hydrolysis^22^. Concurrently, other team members investigated the impact of these molecular weight variations on platelet activation. Our findings revealed significant discrepancies in bleeding time, coagulation time, and platelet activation across gelatin samples with different molecular weights^23^. Informed by the comprehensive literature review, we hypothesized that yak skin gelatin could be employed in the fabrication of scaffold materials. Prior to the fabrication of the yak skin gelatin/PMVE-MA composite scaffold, we optimized the preparation conditions^24^. Subsequently, we prepared yak skin gelatin/PMVE-MA scaffolds using gelatin of distinct molecular weights. Interestingly, the properties of these scaffolds did not correlate linearly with their molecular weights; instead, they predominantly displayed step-like changes in swelling, degradation, platelet adhesion, etc. Notably, the scaffold derived from gelatin with a molecular weight range of 0.1–0.22 μm demonstrated high porosity, a stable degradation rate, and remarkable mechanical strength, with flexural and compressive moduli of 221.500 ± 26.163 and 683.500 ± 62.933 kPa, respectively, alongside superior biocompatibility.

## 2 Materials and Methods

### 2.1 Materials

PMVE-MA and hexamethylene diisocyanate (HMDI) were purchased from Sigma-Aldrich (St. Louis, MO, USA). Phosphate-buffered saline (PBS) was purchased from Beijing Soleibao Technology Co., Ltd. (Beijing, China). Fetal bovine serum (FBS) and minimum essential medium (MEM) basal medium were purchased from Gibco (Life Technologies, California, USA). The penicillin–streptomycin solution was purchased from Wuhan Prosei Biotechnology Co., Ltd. (Wuhan, China). The glutaraldehyde solution (25%) was purchased from Alfa Aesar Chemical Co., Ltd. (Tianjing, China). CCK-8 was purchased from Elabscience Biotechnology Co., Ltd. (Wuhan, China). HepG2 cells were purchased from Pu Nuosai Biotechnology Co., Ltd. (Wuhan, China). The electrophoresis buffer was purchased from Tiangen Biochemical Technology (Beijing, China).

#### 2.1.1 Extraction of Yak Skin Gelatin with Different Molecular Weights

Yak skin was prepared according to the methods described by Chen (2018, 2019).^18,22,25^ Skin was washed and divided into pieces of 30 × 30 cm and then soaked in 2% sodium hydroxide (NaOH) solution for 48 h followed by conducting a dehairing treatment. Soaked and dehaired yak skin was then placed in clean water and further soaked until the pH of the water was neutral, thereby removing any grease adhering to the skin. Then, the skin was washed and stored in a refrigerator at −20 °C.

Alkaline hydrolysis of Yak hides was used to prepare Yak skin gelatin with different molecular weights. The remaining grease was removed from the pretreated Yak skin followed by cutting the skin into pieces of 5 × 5 cm. Pieces were then heated in distilled water with a ratio of 1:10 (w/v) at 80 °C for 6 h, stirring continuously. This heating process was repeated twice followed by filtering and combining the resulting gelatin solution. The Yak skin gelatin solution was then applied through different filter diameters (0.22 and 0.1 μm) using ultrafiltration membranes to obtain four Yak skin gelatin solution samples, i.e., A, B, C, and D. Then, these four samples were freeze-dried for use in future experiments.

#### 2.1.2 Sodium Dodecyl Sulfate–Polyacrylamide Gel Electrophoresis (SDS-PAGE)

Electrophoresis was conducted by using the sodium dodecyl sulfate–polyacrylamide gel electrophoresis (SDS-PAGE) system, according to the method of Lawrence and Besir (2009), to determine the molecular weight range of the yak skin gelatin^26^. Gelatin samples were dissolved with 0.5 mol/L acetic acid to a 2 mg/mL sample solution, mixed with a protein loading buffer, heated until the protein was fully denatured, and stored at 4 °C for later use. Preparation of 12% separating gel and 5% concentrated gel was done as summarized in Table 1, with a sample loading volume of 7 μL. Constant pressure electrophoresis at 90 V for 105 min was conducted, waiting until the front edge of the indicator moved down to the lower edge of the glass plate and then stopping electrophoresis. After electrophoresis was stopped, staining was conducted in a solution of ethanol, ultrapure water, and glacial acetic acid (9:9:2) and 2.5 g/L Coomassie Brilliant Blue (R-250) for 1 h. After staining, a mixed solution of ethanol, glacial acetic acid, and ultrapure water (1:2:17) was used, which decolorized the solution until the protein bands were clear.

**Table 1:** Formulations of the 12% Separation Gel and 5% Spacer Gel

	**12% separation gel**	**5% spacer gel**
ddH_2_O	3.4	2.0
30% Acr-Bis (29:1)	4.0	1.0
gel buffer A	2.5	
gel buffer B		3.0
10% APS	0.1	0.06
TEMED	0.006	0.006

### 2.2 Experimental Animals

Due to male rats having more blood reserves, 20 male Sprague–Dawley (SD) rats (180–220 g) were procured from the Experimental Animal Centre of Gansu University of Chinese Medicine (Gansu, China; Certificate No. SCXK 2015-0001). All animals were kept in a temperature-controlled environment (25 ± 2 °C, 55 ± 5% relative humidity, and a 12 h light–dark cycle), had unrestricted access to food and water, and were allowed to adapt to their environment for at least 1 week before any experiment. Before experiments, all animals were fasted for 12 h.

All animal procedures were conducted in accordance with the WHO Guidance of Humane Care and Use of Laboratory Animals^27^. All animal procedures were performed in accordance with the Guidelines for Care and Use of Laboratory Animals of the Northwest Institute of Plateau Biology and approved by the Animal Ethics Committee of the Northwest Institute of Plateau Biology.

### 2.3 Methods

#### 2.3.1 Fourier-Transform Infrared Spectroscopy (FTIR) Analysis

An appropriate amount of gelatin was weighed to different molecular weights and then analyzed using a Fourier transform infrared spectrometer (FTIR) attenuated total reflection method to determine the scanning range of 4000–400 cm^–1^^28^.

#### 2.3.2 Atomic Force Microscopy (AFM) Analysis

The surface morphology of gelatin with different molecular weights of Yak skin was observed with an AFM. The scan area size of the sample was 3 × 3 μm.

#### 2.3.3 Amino Acid Detection and Analysis

Gelatin samples were weighed, placed into an anaerobic hydrolysis tube with 5 mL of 6 N hydrochloric acid added, and mixed. They were then put into a refrigerant, i.e., liquid nitrogen or dry ice, and frozen. After the solution in each sample had solidified, it was removed and then vacuum sealed through the suction tube of the vacuum pump. The tube was then hydrolyzed in a constant temperature drying box, at 110 °C for 24 h, with the volume fixed to 10 mL after cooling. A 0.45 μm water-based filter membrane was used to remove impurities. A 0.5 mL amount of the filtrate was then placed in an Eppendorf (EP) tube and vacuum-dried in a vacuum concentrator. The substance was dissolved in 1 mL of deionized water and then dried; this was repeated twice. Finally, 1 mL of a pH 2.2 sample diluent was added to dissolve, filtered with a 0.22 μm aqueous membrane, and analyzed using an automatic amino acid analyzer^29^.

#### 2.3.4 Differential Scanning Calorimeter (DSC) Analysis

A 5 mg amount of gelatin samples was weighed in a DSC crucible, sealed, and tested for thermal stability. Using an empty crucible as a reference, the scanning range was determined to be 25–250 °C, with the heating rate at 5 °C/min and the nitrogen flow rate in the sample chamber at 20 mL/min^30^.

#### 2.3.5 Preparation of the Gelatin/PMVE-MA Composite Scaffold Material

Using 0.5 mol/L acetic acid solution for dissolving, 8% w/v molecular weights were prepared for the different samples: (A) <0.1 μm yak skin gelatin, (B) 0.1–0.22 μm yak skin gelatin, (C) >0.22 μm yak skin gelatin, and (D) full range yak skin gelatin. Similarly, a 1.33% w/v PMVE-MA solution was dissolved and prepared. The two solutions were mixed in a ratio of 1:1 and magnetically stirred for 3.85 h at a stirring temperature of 43 °C. After the stirring process, the mixed solution was poured into a suitable mold, frozen at −80 °C for 24 h, and then freeze-dried. The freeze-dried material sample was soaked in a 10% hexamethylene diisocyanate (HMDI) solution prepared with isopropyl alcohol for a certain period of time and then washed with isopropanol twice. After that, isopropanol was left to evaporate naturally and then washed with pure water three times.^24,31^

#### 2.3.6 Preparation of an Extract of a Gelatin/PMVE-MA Composite Scaffold Material

As per methods outlined in the literature^32^, the composite scaffold material was immersed in 75% ethanol for 2 h and sterilized by UV irradiation for 2 h. After the ethanol was left to evaporate naturally, the material was put into a sterile centrifuge tube. The test tube contained 10% FBS and 1% penicillin–streptomycin MEM. Material extracts were prepared according to the ratio of 50 g of material to 1 L of MEM culture solution, then the material and culture solution were placed into a 37 °C incubator for 48 h. A 0.22 μm filter was used for extract. After filtering with a microporous filter, it was placed in a sterile centrifuge tube to obtain a composite scaffold material extract with a concentration of 50 g/L, which was then stored in a refrigerator at 4 °C for later use.

#### 2.3.7 Scaffold Morphology analysis

The prepared composite scaffold material was placed in an ion sputtering apparatus. Platinum was sprayed to coat the surface of the material with a platinum film and then placed under a scanning electron microscope (SEM) to observe the surface morphology of the material^33^.

#### 2.3.8 Swelling Studies

The water absorption capacity of the scaffold material was evaluated by measuring the swelling degree of the stent material. The dry scaffold material was weighed and recorded (Wd), soaked in a pH 7.4 PBS solution at room temperature, and immersed for 1, 3, 5, 10, and 24 h. Following this, the soaked scaffold material was removed at 48 h, the remaining PBS on the surface of the material was absorbed with filter paper, and the weight was recorded (Ww)^34^. The following swelling degree calculation formula was used:
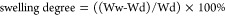


#### 2.3.9 Degradation Analysis

The dried stent material was weighed and recorded (Wd) and then soaked in a pH 7.4 PBS solution at room temperature and 37 °C for 14 consecutive days. The PBS solution was changed every day, with the stent material changed after 14 days; it was then freeze-dried, weighed, and recorded (Wt)^35^. The following degradation rate calculation formula was used:
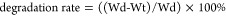


#### 2.3.10 Porosity of the Composite Scaffolds

The ethanol substitution method was used to test the porosity of the scaffold material to determine the volume of the scaffold material (*V*) and the mass (W1). The scaffold material was soaked in a certain volume of absolute ethanol; the material was removed after it was completely saturated. After the surface liquid was absorbed, the material was then weighed (W2). The density of absolute ethanol was recorded as ρ^36^. The porosity of the scaffold material is denoted as *P*, with the following calculation formula used:
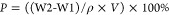


#### 2.3.11 Mechanical Characterization

The method according to Xiaotong et al. (2017) was used, i.e., GB/T 1448-2005, the universal electronic testing machine to measure the compressive strength of the composite scaffold material^37^. The composite scaffold material was then cut into a square block of ∼10 × 10 mm and fixed in the device at a rate of 10 mm/min.

The universal electronic testing machine was used to measure the bending strength of the composite stent material. The composite scaffold material was then cut into a rectangular parallelepiped shape of 18 × 5 × 4 mm and fixed to the device, and then the strength was measured at a rate of 5 mm/min.

#### 2.3.12 Hemolysis Analysis

Based on the method of Javanmard et al. (2016), a hemolysis experiment was carried out^38^. Whole blood samples of healthy SD rats were placed in an anticoagulation blood collection tube and immediately centrifuged at 4 °C and 3000 rpm for 15 min. The lower layer of red blood cells was then washed with normal saline until a clear solution was obtained. Then, the clear red blood cells were diluted with normal saline to a 2% red blood cell solution (1 mL of red blood cells + 49 mL of normal saline). The scaffold material was placed on a 24-well plate. A 1 mL amount of normal saline was added to each well, with the scaffold kept for 30 min at 37 °C in the water bath. The saline was then discarded, and the diluted red blood cell solution was added to the scaffold in a 37 °C water bath. After incubation in the medium for 1 h, the incubated red blood cell solution was transferred to a 2 mL centrifuge tube and centrifuged at 4 °C and at 1500 rpm for 15 min. The supernatant was then placed in a 96-well plate (100 μL/well), with the OD value read at 545 nm, and the hemolysis rate was consequently calculated. The positive control was served by the coincubation of pure water and red blood cell solution, while the negative control was the coincubation of normal saline and red blood cells. The hemolysis rate was calculated as follows:
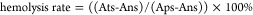
where ts is the test sample, ns is the negative control, and ps is the positive control.

#### 2.3.13 Platelet Adhesion Assay

The platelet adhesion amount was determined according to the method of Javanmard et al. (2016)^38^. Whole blood was obtained from healthy SD rats, with platelet-rich plasma obtained through centrifugation. The scaffold material was placed in a 24-well plate with PBS in a 37 °C water bath. The material was balanced for 30 min, PBS was discarded, and 1 mL of platelet-rich plasma was added per well in a 37 °C water bath. They were then incubated for 30, 60, and 120 min, and the suspension was aspirated and washed in PBS to remove unadherent blood sizes.

#### 2.3.14 Cell Proliferation Assay

HepG2 cells were cultured in the following conditions: 37 °C, 5% CO_2_, in a MEM containing 10% FBS and 1% penicillin-streptomycin, and a degree of cell fusion. When cells reached more than 80%, cell passaging and cocultivation of cells and materials were performed.

Cell proliferation experiments of composite materials with different gelatin components were divided into four groups. Experiment 1 was the positive control group with cells containing the MEM complete medium; experiment 2 was the material extract low concentration group (12.5 mg/mL); experiment group 3 was the material extract medium concentration group (25 mg/mL); and experiment group 4 was the material extract high concentration group (50 mg/mL). Groups 1, 2, 3, and 4 were placed in the extract liquid for administration at 2, 4, 6, and 8 days, respectively. Cell proliferation was detected after 2, 4, 6, and 8 days. The CCK-8 method was used to study the effect of the scaffold material extract on cell proliferation.

Cell suspension was inoculated with a density of 25,000 cells/mL (100 μL/well) in a 96-well plate and cultured in a CO_2_ incubator for 24 h. After the cells adhered to the wall, the original culture medium was discarded. Cultures were washed twice with PBS, and MEM was added to complete the medium (to serve as the positive control group). Material from each concentration group of A, B, C, and/or D was extracted at 100 μL and placed into a CO_2_ incubator to continue culturing for 2, 4, 6, and 8 days, respectively. After 8 days, the corresponding culture plate was removed and replaced with MEM, and 10 μL of CCK-8 solution was added to each well, shaken for 1 min in a microplate shaker, and incubated in a CO_2_ incubator for 2 h to fully react. The OD value of each group was measured at 450 nm with a microplate reader. All data were recorded for each group, with the relative growth calculated for each group of cells. For each group, six samples were tested in parallel, with the average value recorded. The following calculation formula for the relative appreciation rate was used:
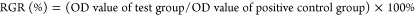


#### 2.3.15 Cell Adhesion Assay

According to Williamson et al. (2006), each group of scaffold materials was placed on a 24-well cell culture plate, inoculating the surface of the HepG2 cell scaffold material with a cell density of 2.5 × 10^4^ cells/mL (1 mL/well)^39^. Groups were then incubated in a CO_2_ incubator for a certain period of time and washed with PBS. The scaffold material cocultured with cells was used twice to remove the cells that were not adhered to the scaffold material. The adhered cells were then fixed with 2.5% glutaraldehyde at room temperature for 2 h, with the cells being fixed overnight at 4 °C. Before observing cell adhesion through SEM, the material sample was dehydrated with graded ethanol and evaporated until dry. The pretreated cell–material composite was placed in an ion sputtering apparatus, with the surface of the material plated with platinum. The membrane was placed under SEM to observe the surface morphology of the material and the adhesion of cells.

#### 2.3.16 Cell Infiltration Assay

The cell infiltration experiment uses HepG2 cells with green fluorescent protein (GFP) for easy observation. Each group of scaffold materials was placed on a 24-well cell culture plate, with the surface of HepG2 cell scaffold materials inoculated with a cell density of 2.5 × 10^4^ cells/mL (1 mL/well), and incubated in a CO_2_ incubator for a certain period of time. The node removes the cell–material complex and observes the growth and infiltration of cells in the scaffold material through a laser confocal microscope. In the *z* dimension, every 2 μm is taken to evaluate the cell growth inside the scaffold material^31^.

### 2.4 Statistical Analysis

The experimental data were analyzed and processed using Excel 2007, GraphPad Prism 7, and Origin 18.0. Single-factor analysis of variance was used to evaluate the statistical significance of the data, defined as *p < 0.05 (GraphPad Prism 7). The SDS-PAGE images were photographed using a gel imaging system, and the AFM imaging data were analyzed offline using the Nanoscope Analysis software.

#### Ethical Approval

Ethical approval for animal experiments was obtained from the Laboratory Animal Ethics Committee, Northwest Institute of Plateau Biology, Chinese Academy of Sciences.

## 3 Results

### 3.1 Physicochemical Properties of Yak Skin Gelatin with Different Molecular Weights

#### 3.1.1 SDS-PAGE

**Figure 1:**
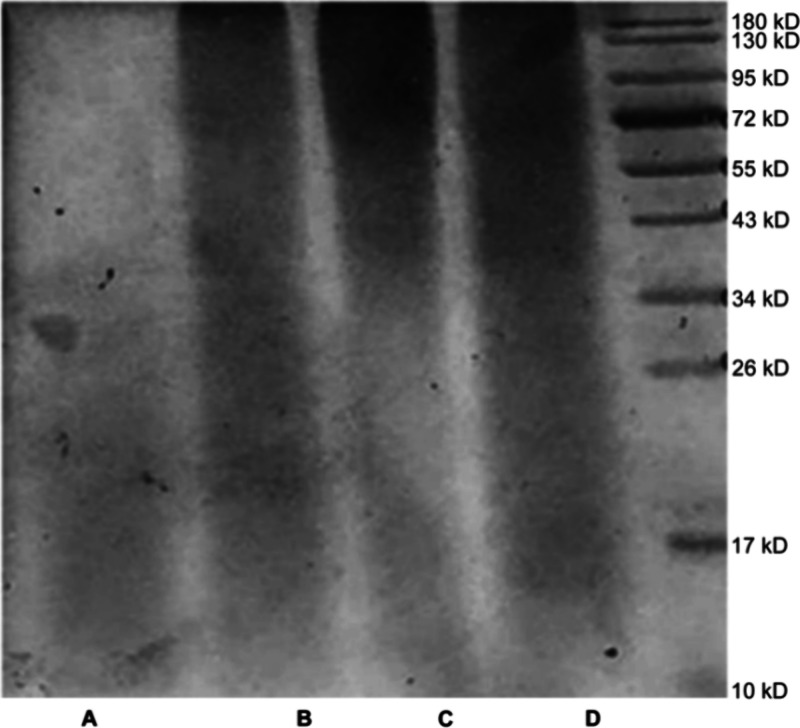
SDS-PAGE patterns of yak skin gelatin with different molecular weights (M: molecular weight marker; A: yak skin gelatin below 0.1 μm; B: yak skin gelatin between 0.1 and 0.22 μm; C: yak skin gelatin above 0.22 μm; and D: whole yak skin gelatin).

Figure 1 illustrates the SDS-PAGE spectrum of yak hide rubber in the molecular weight range of 10–180 kDa. It was found that the molecular weight of A was mainly distributed in the range of 10–26 kDa, the molecular weight of B was mainly distributed in the range of 17–43 kDa, the molecular weight of C was mainly distributed in the range of 43–180 kDa, and the molecular weight of D was mainly distributed in the range of 17–180 kDa.

#### 3.1.2 FTIR Spectroscopy Analysis

**Figure 2:**
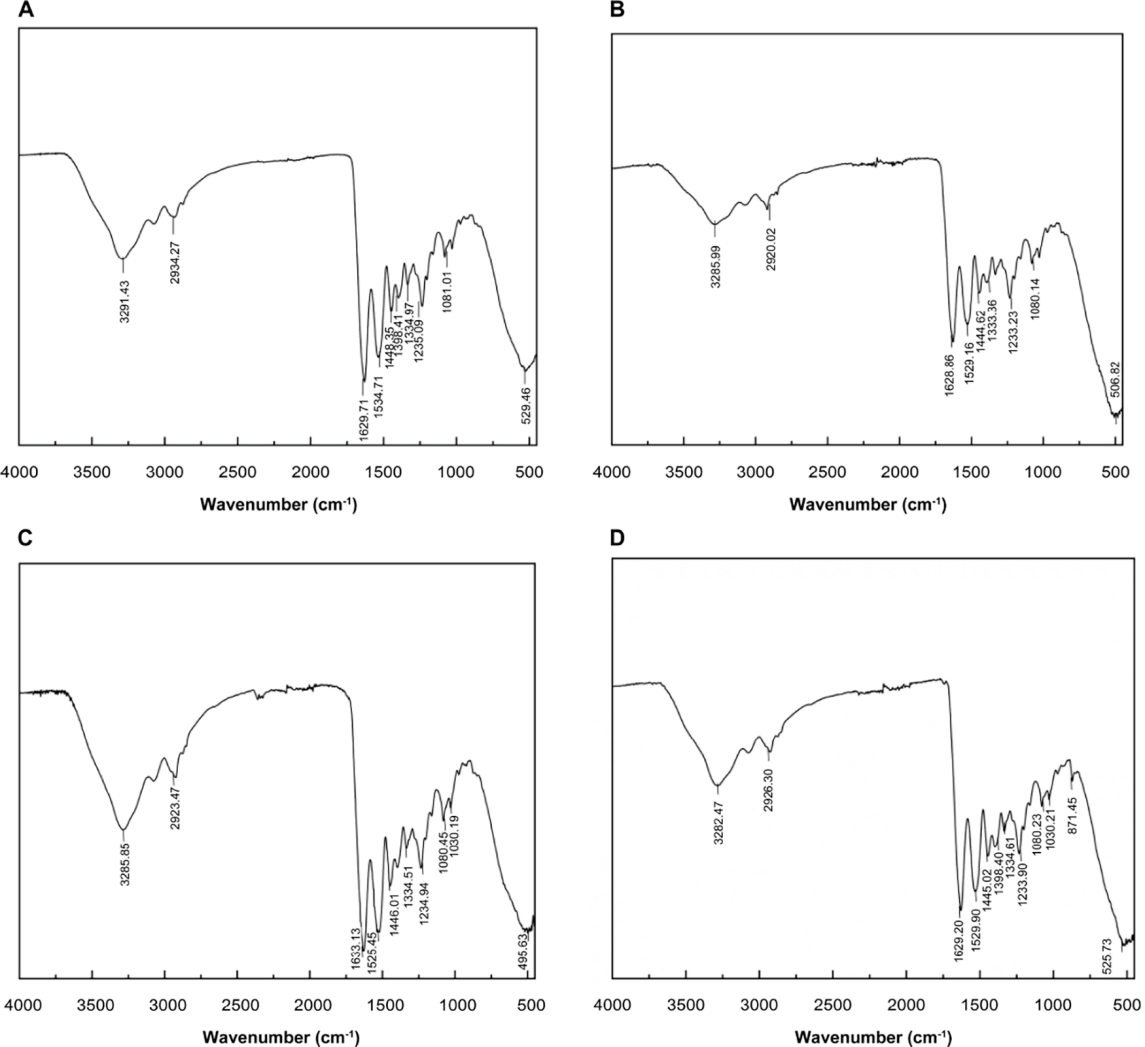
FTIR spectra of yak skin gelatin with different molecular weights (A: yak skin gelatin below 0.1 μm; B: yak skin gelatin between 0.1 and 0.22 μm; C: yak skin gelatin above 0.22 μm; and D: whole yak skin gelatin).

The results of the FTIR analysis of yak skin gelatin with different molecular weights are illustrated in Figure 2. The characteristic absorption peaks of the amide A band were observed at 3291.43 (A), 3285.99 (B), 3285.85 (C), and 3282.47 cm^–1^ (D), which represent OH or NH stretching vibration^40^. The characteristic absorption peaks of the amide B band (3080–3100 cm^–1^) were observed at 2934.27 (A), 2920.02 (B), 2923.47 (C), and 2926.30 cm^–1^ (D); the absorption peak was related to the asymmetric stretching of the CH_2_ group^41^. Characteristic absorption peaks of gelatin in the amide I band (1600–1660 cm^–1^) of each molecular weight range were located at 1629.71 (A), 1628.86 (B), 1633.13 (C), and 1,629.20 cm^–1^ (D). The amide I band is the C=O stretching vibration characteristic band; the absorption peak in this region can reflect changes in the secondary structure of a protein; thus, the amide I band is often used to analyze protein secondary structure^42^. The amide II band (1500–1600 cm^–1^) exhibits NH bond bending and CN bond stretch^43^. The peak positions of different molecular weight gelatin in the amide II band were observed at 1534.71 (A), 1529.16 (B), 1525.45 (C), and 1529.90 cm^–1^ (D). The characteristic absorption peaks of the amide III band of different molecular weight gelatin are located at 1235.09 (A), 1233.23 (B), 1234.94 (C), and 1233.90 cm^–1^ (D); the amide III band is affected by the side chain group, with a variety of deformation vibration modes^44^.

#### 3.1.3 AFM Analysis

**Figure 3:**
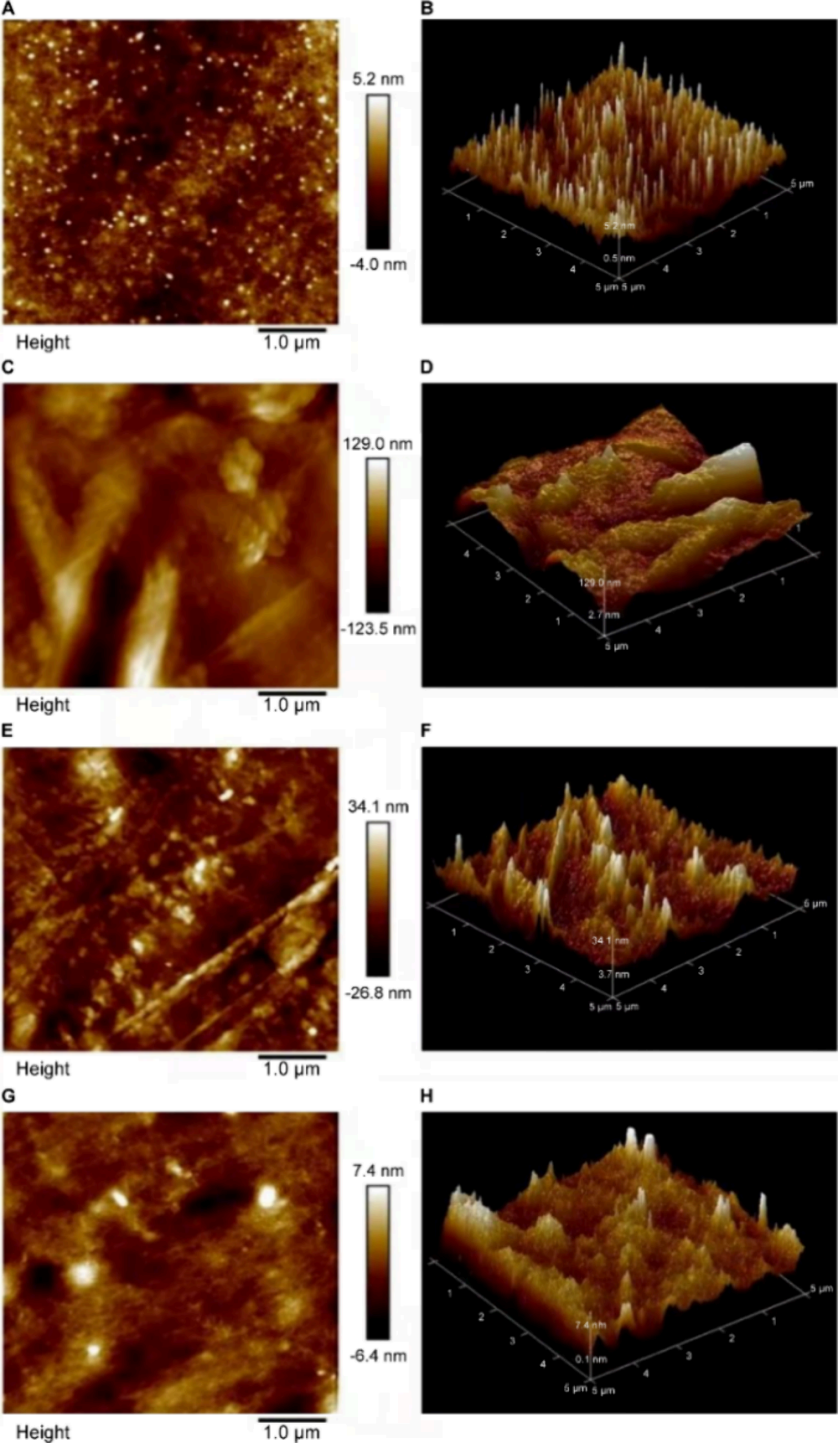
AFM images of yak skin gelatin with different molecular weights (A and B: yak skin gelatin below 0.1 μm; C and D: yak skin gelatin between 0.1 and 0.22 μm; E and F: yak skin gelatin above 0.22 μm; G and H: whole yak skin gelatin).

Figure 3 illustrates the morphological structure of different molecular weights of gelatin with images of the surface as well as 3D images of each gelatin molecular weight. Table 2 shows the roughness and the average height. The yak skin gelatin roughness was 48.40 nm, with an average height at 14.622 nm of the B group gelatin. Its roughness and average height were the highest among all samples. Group A had the smallest molecular weight, and its roughness and average height were also low.

**Table 2:** Roughness and Average Height of Yak Skin Gelatin^a^

**sample**	**roughness (nm)**	**average height (nm)**
A	0.93	2.697
B	48.40	14.622
C	9.00	6.132
D	1.19	2.354

a A: yak skin gelatin below 0.1 μm; B: yak skin gelatin between 0.1 and 0.22 μm; C: yak skin gelatin above 0.22 μm; and D: whole yak skin gelatin.

Combining the AFM images of each group of gelatins showed that group B had a roughness of 48.40 nm and an average height of 14.622 nm, indicating that its roughness and average height were the highest among all of the groups. Group A had the smallest molecular weight, and its roughness and average height were also low. The results showed that gelatins with different molecular weights have different roughness and mean height, and the correlation parameters vary with the molecular weight range, but their roughness and mean height have no correlation with the molecular weight range.

#### 3.1.4 Amino Acid Analysis

The nutrition and function of proteins are mainly determined by the type and quantity of amino acids. The amino acid composition and content of different molecular weights of yak skin gelatin are shown in Table 3.

**Table 3:** Amino Acid Composition and Content of Yak Skin Gelatin^a^

**amino acid**	**content** (mg/g)				**percentage (%)**			
	**A**	**B**	**C**	**D**	**A**	**B**	**C**	**D**
Asp	53.849	55.412	52.735	55.316	7.171	6.311	6.308	6.362
Thr	15.807	15.673	14.685	15.285	1.926	1.757	1.757	1.758
Ser	25.861	25.194	23.535	24.582	3.151	2.869	2.815	2.827
Glu	91.453	96.376	91.612	95.266	11.144	10.977	10.959	10.957
Gly	198.503	217.228	208.914	217.719	24.188	24.741	24.992	25.042
Ala	79.157	102.690	97.755	101.483	9.645	11.696	11.694	11.673
Cys	3.651	2.444	0.652	1.316	0.445	0.278	0.078	0.151
Val	23.361	22.546	20.229	22.814	2.847	2.568	2.420	2.624
Met	5.613	7.454	6.943	7.398	0.684	0.849	0.831	0.851
Ile	14.761	13.872	13.677	14.562	1.799	1.580	1.636	1.675
Leu	30.458	28.905	27.450	29.026	3.711	3.292	3.284	3.339
Tyr	8.661	5.987	5.518	6.087	1.055	0.682	0.660	0.700
Phe	18.956	18.572	17.496	18.898	2.310	2.115	2.093	2.174
His	25.222	23.850	24.387	25.735	3.073	2.716	2.917	2.960
Lys	26.047	35.482	34.249	35.206	3.174	4.041	4.097	4.049
Arg	72.106	79.562	75.089	78.375	8.786	9.062	14.476	9.015
Pro	127.207	126.752	121.012	120.348	15.500	14.436	14.476	13.842
total	820.672	878.001	835.937	869.418	100.000	100.000	100.000	100.000

a A: yak skin gelatin below 0.1 μm; B: yak skin gelatin between 0.1–0.22 μm; C: yak skin gelatin above 0.22 μm; and D: whole yak skin gelatin.

Among different molecular weight segments of gelatin, the amount of glycine was the highest followed by proline, accounting for ∼25 and ∼14% of the total amino acid content, respectively. The total amount of gelatin amino acids was the highest in group B followed by group D, and group A had the lowest amount of gelatin amino acids. The amount of essential amino acids (lys, trp, phe, met, thr, ile, leu, and val) was the highest in group D followed by group B. The amounts of essential amino acids in groups A and C were significantly lower than those in groups B and D.

#### 3.1.5 DSC Analysis

**Figure 4:**
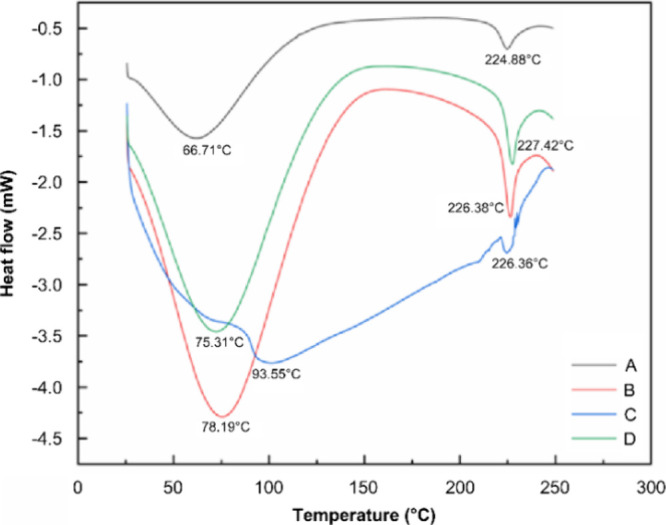
DSC of Yak skin gelatin with different molecular weights (A: yak skin gelatin below 0.1 μm; B: yak skin gelatin between 0.1 and 0.22 μm; C: yak skin gelatin above 0.22 μm; and D: whole yak skin gelatin).

Through the DSC analysis of yak skin gelatin, thermodynamic properties were obtained (Figure 4). It can be seen from the figure that when the temperature is increased from 25 to 250 °C, the heat absorption peak shows the destruction of the sample and the change of conformation. The first absorption peak in the figure is related to the heat shrinkage temperature: with heat shrinkage temperatures of A, B, C, and D at 66.71, 78.19, 93.55, and 75.31 °C, respectively. The second peak is related to the degradation of polymer chains, and the heat shrinkage process leads to the structure of gelatin damaged and decomposed when the crystals are broken and melted. The melting temperatures of collagen types A, B, C, and D were 224.88, 226.38, 226.36, and 227.42 °C, respectively. The above results show that the thermodynamic properties of gelatin in different molecular weight ranges are different. Among them, the gelatin of >0.22 μm has the highest heat shrinkage temperature, and the melting temperature of the gelatin of each molecular weight range is higher than 200 °C. The results show that the thermal stability of yak skin gelatin is higher than that of other mammals^45,46^ and that they possess great potential for development.

### 3.2 Physical Properties of Composite Scaffold Materials

#### 3.2.1 Scaffold Morphology Analysis

**Figure 5:**
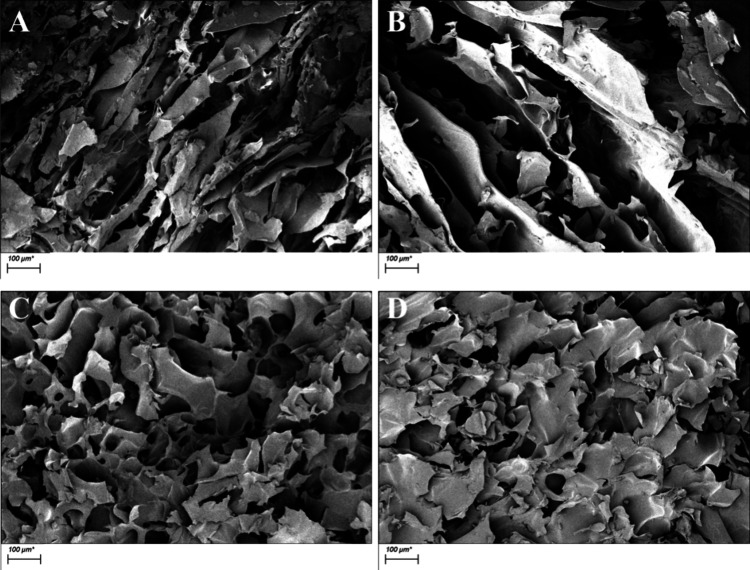
SEM micrograph of scaffold materials (100×) (A: <0.1 μm yak skin gelatin/PMVE-MA composite scaffold materials; B: 0.1–0.22 μm yak skin gelatin/PMVE-MA composite scaffold materials; C: >0.22 μm yak skin gelatin/PMVE-MA composite scaffold materials; and D: yak skin gelatin/PMVE-MA composite scaffold materials).

The microstructure of the gelatin/PMVE-MA composite support material is illustrated in Figure 5. Group A has irregular pore structure and a disordered sheet structure; the group B material has a loose sheet structure, while the surface structure of group C is close to that of group D, with irregular pore structure and denser than that of group B. Group A has similar structures with B, but its porosity is lower than group B. Groups C and D have similar structures with a certain pore structure, but each hole in the structure forms an independent unit that is isolated from each other. Consequently, cells within these isolated pores experience diminished intercellular interactions and communication, dense structure, and less communication between pores. In contrast, group B has larger pores in scale and is to some extent interconnected with other pores, providing more opportunities for cell communication and further accelerating the process of re-endothelialization.

#### 3.2.2 Swelling Studies

**Figure 6:**
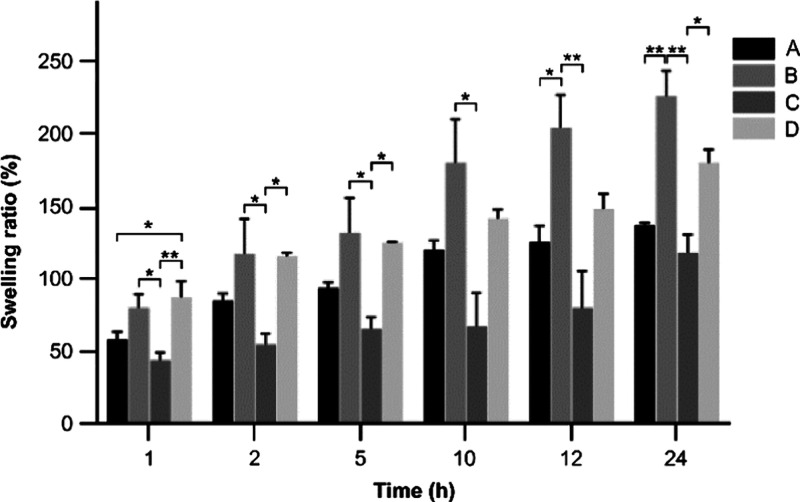
Changes of the swelling ratio of composite scaffolds at different times (A: <0.1 μm yak skin gelatin/PMVE-MA composite scaffold materials; B: 0.1–0.22 μm yak skin gelatin/PMVE-MA composite scaffold materials; C: >0.22 μm yak skin gelatin/PMVE-MA composite scaffold materials; D: yak skin gelatin/PMVE-MA composite scaffold materials; compared with each composite scaffold material at the same time: *P < 0.05 and **P < 0.01).

Swelling degree is an important characteristic of scaffold materials, and good swelling performance is conducive to cell adhesion and proliferation on scaffold materials. Figure 6 shows the change of swelling degree of different molecular weight gelatin/PMVE-MA composite scaffold materials after soaking in PBS solution for 1, 2, 5, 10, 12, and 24 h. It can be seen from the figure that the scaffold material swells rapidly within 5 h; 10 h later, the swelling degree of the stent material reaches relative equilibrium. The swelling degree of the stent material depends on the hydrophilicity of the polymer and the microstructure of the stent material. Because the polymer PMVE-MA has a good hydrophilicity, the stent material also shows good hydrophilicity. By comparison of the results, it can be seen that the swelling degree of the material in group B is the highest, while that of group C is the lowest. Combined with the observation results of the microstructure of different composite scaffold materials, the materials of group B have many pore structures and are connected to each other, which can promote the swelling of the materials. Therefore, the swelling degree of the materials in group B is the highest. The observation results of the microstructure of the other three groups of materials show a lamellar structure with messy and irregular pores, so the swelling degree is lower than that of group B materials.

#### 3.2.3 Degradation Analysis

**Figure 7:**
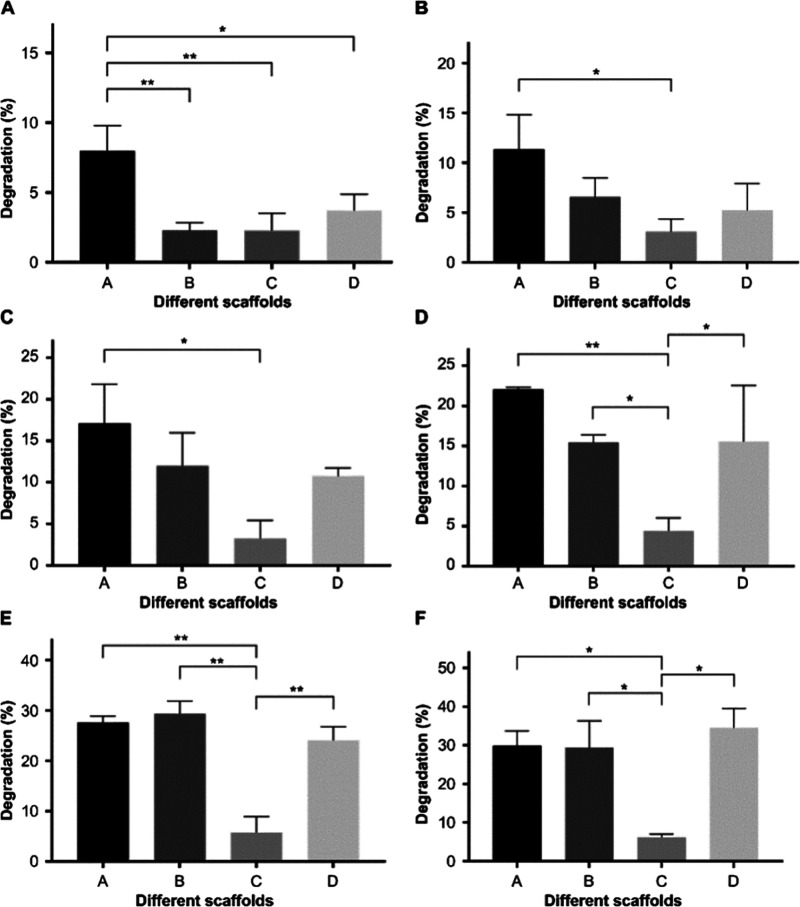
Changes in the degradation ratio of composite scaffolds at different times (A: <0.1 μm yak skin gelatin/PMVE-MA composite scaffold materials; B: 0.1–0.22 μm yak skin gelatin/PMVE-MA composite scaffold materials; C: >0.22 μm yak skin gelatin/PMVE-MA composite scaffold materials; and D: yak skin gelatin/PMVE-MA composite scaffold materials). Panels A–F show the degradation times of 1, 3, 5, 7, 10, and 14 days. Compared with each group: *P < 0.05 and **P < 0.01.

Figure 7 shows the mass loss rate of different molecular weight gelatin/PMVE-MA composite scaffold materials after immersing in PBS solution for 1, 3, 5, 7, 10, and 14 days under a 37 °C water bath. The degradation rate of the material gradually increased over time. After day 1 of degradation, the degradation rate of group A materials was significantly higher than those of the other three groups of materials. At day 3 and day 5 of degradation, the degradation rate of group A materials was significantly higher than that of group C materials. Further, there was no significant difference in the degradation rate of the other groups. At day 7 of degradation, the degradation rate of group C materials is extremely different from that of group A, and it is significantly different from those of groups B and D. After day 10, the degradation rate of group C materials was significantly lower than those of groups A, B, and D. At day 14 of degradation, the degradation rate of group C was significantly lower than those of groups A, B, and D. At each time point, the degradation rate of group C was the lowest. Combining the results of the material microstructure and swelling degree, group C had a compact structure and the lowest swelling degree, so the degree of degradation was also the lowest. After 14 days of degradation at 37 °C, the retention rate of the C group material still reached more than 90%, and the retention rates of the other groups were all at ∼70%, meaning that the four material groups can meet the requirements of the scaffold material into the body.

#### 3.2.4 Porosity of the Composite Scaffolds

Porosity results of different molecular weight gelatin/PMVE-MA composite scaffold materials are shown in Table 4. It can be seen from the table that the porosity of the composite scaffold material is related to the molecular weight of the gelatin. Group A has the lowest porosity at ∼65%. As the molecular weight of gelatin increased, the porosity of the composite scaffold also increased. The porosity of group D was as high as 90%, but the porosity of each group of materials did not change significantly with molecular weight, and there was no significant difference between each group. With the exception of group A, the porosity of the groups was between 70 and 90%, which can provide enough growth space for cells.^47,48^

**Table 4:** Porosity of the Different Composite Scaffolds^a^

**scaffold**	**porosity (%)**
A	64.920 ± 6.530
B	87.317 ± 8.332
C	81.315 ± 12.606
D	90.564 ± 7.474

a A: <0.1 μm yak skin gelatin/PMVE-MA composite scaffold materials; B: 0.1–0.22 μm yak skin gelatin/PMVE-MA composite scaffold materials; C: >0.22 μm yak skin gelatin/PMVE-MA composite scaffold materials; and D: yak skin gelatin/PMVE-MA composite scaffold materials.

#### 3.2.5 Mechanical Characterization

**Figure 8:**
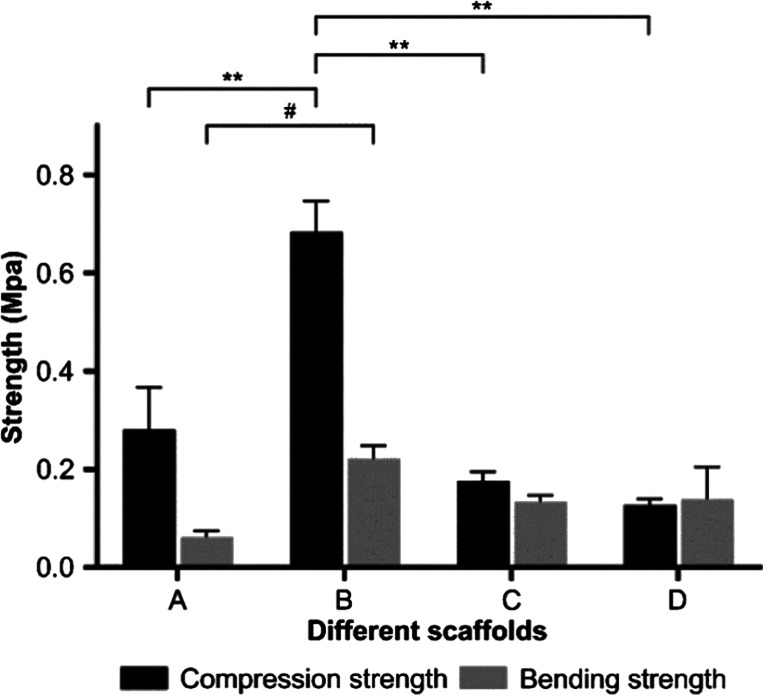
Mechanical strength of different scaffold materials (compared with group B material: *P < 0.05 and **P < 0.01; A: <0.1 μm yak skin gelatin/PMVE-MA composite scaffold materials; B: 0.1–0.22 μm yak skin gelatin/PMVE-MA composite scaffold materials; C: >0.22 μm yak skin gelatin/PMVE-MA composite scaffold materials; and D: yak skin gelatin/PMVE-MA composite scaffold materials).

Figure 8 illustrates the mechanical properties of different molecular weight gelatin/PMVE-MA composite scaffold materials. The results show that the compressive strength of group B was significantly higher those of the other three groups, and the bending strength of group B materials was also significantly higher than that of group A. This indicates that the mechanical properties of group B were significantly better than the other three groups, with their bending strength and compressive strength being 221.500 ± 26.163 and 683.500 ± 62.933 kPa, respectively. The compressive strength here was higher than that of the vascular stent material prepared by Zhu et al.^36^, which can meet the requirements of vascular stent materials in vivo.

### 3.3 Hemocompatibility of Composite Scaffold Materials

#### 3.3.1 Platelet Adhesion Analysis

**Figure 9:**
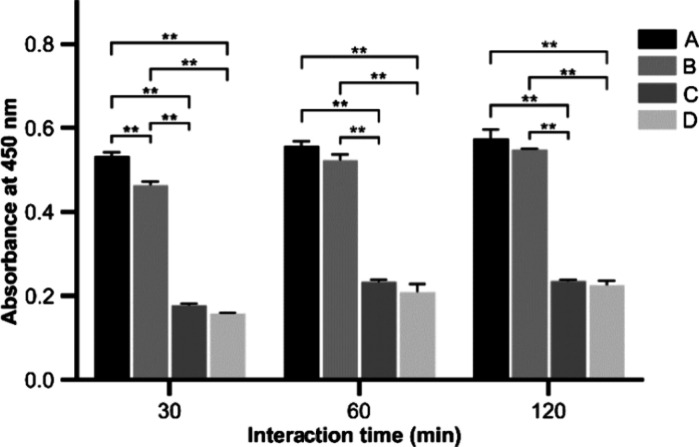
Determination of platelet adhesion on composite scaffolds (A: <0.1 μm yak skin gelatin/PMVE-MA composite scaffold materials; B: 0.1–0.22 μm yak skin gelatin/PMVE-MA composite scaffold materials; C: >0.22 μm yak skin gelatin/PMVE-MA composite scaffold materials; and D: yak skin gelatin/PMVE-MA composite scaffold materials; compared with the individual scaffold material concentrations within the group: *P < 0.05 and **P < 0.01).

Platelet adhesion on the scaffold surface was evaluated by LDH quantification. LDH activity is positively associated linearly with the platelet number, so it is reliable to detect platelet content by LDH quantification.^49,50^ As illustrated in Figure 9, platelet adhesion on the composite scaffold material increased slightly with the incubation time; when the incubation time was 30 min, the platelets of group A were significantly higher than those of groups B, C, and D at 60 and 120 min. In addition, the amount of platelet adhesion was related to the molecular weight of gelatin: the larger the molecular weight, the less the platelet adhesion amount. According to the figure, the platelet adhesion amount of group D materials was the lowest.

#### 3.3.2 Hemolysis Analysis

**Figure 10:**
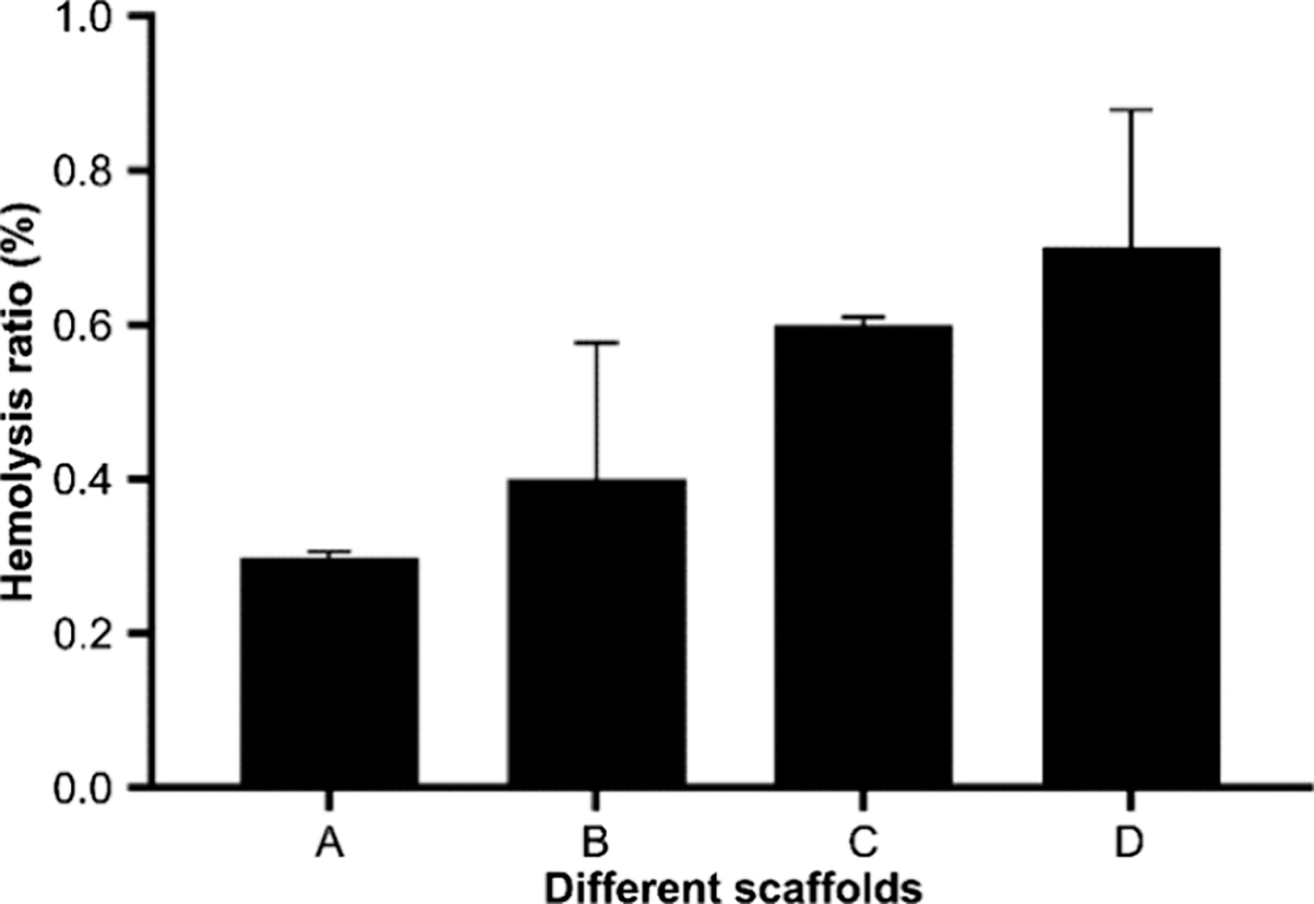
Hemolysis ratio of different scaffold materials (A: <0.1 μm yak skin gelatin/PMVE-MA composite scaffold materials; B: 0.1–0.22 μm yak skin gelatin/PMVE-MA composite scaffold materials; C: >0.22 μm yak skin gelatin/PMVE-MA composite scaffold materials; and D: yak skin gelatin/PMVE-MA composite scaffold materials).

The effect of gelatins with different molecular weights on the hemolysis rate of composite materials is illustrated in Figure 10. The hemolysis rate of composite materials increases with an increase in the molecular weight of gelatin. The hemolysis rate for the whole gel/PMVE-MA material was the highest, but the hemolysis rate of the four materials was less than 1% for all of them. There was no significant difference between the hemolysis rates of each group of materials, indicating that the molecular weight of gelatin has no obvious influence on the hemolysis rate of composite stent materials. The hemolysis rate of all composite stent materials is less than 5%, which can meet the requirements of blood vessels for stent materials required to enter the body^51^.

### 3.4 Cytocompatibility of Composite Scaffold Materials

#### 3.4.1 Cell Proliferation

**Figure 11:**
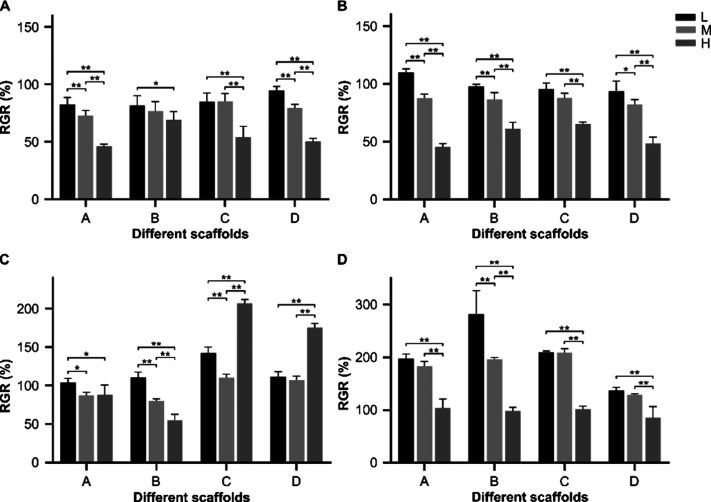
Effect of extracts from different scaffold materials on cell proliferation (A: <0.1 μm yak skin gelatin/PMVE-MA composite scaffold materials; B: 0.1–0.22 μm yak skin gelatin/PMVE-MA composite scaffold materials; C:> 0.22 μm yak skin gelatin/PMVE-MA composite scaffold materials; and D: yak skin gelatin/PMVE-MA composite scaffold materials; compared with the individual administered concentrations within the group: *P < 0.05 and **P < 0.01).

Figure 11 shows the effect of different molecular weight gelatin/PMVE-MA composite scaffold extracts on cell proliferation. Results showed that with the extension of the culture time, cell activity gradually increased, indicating that the proliferation of cells in the positive control group and the experimental group of different extracts of different concentrations was improving. The extract was administered for 2 and 4 days. The cell proliferation rate of the high concentration group was significantly lower than that of the low concentration group. After 6 days, the cell proliferation rate of the high concentration extract of groups C and D was significantly higher than that of the other concentration groups. After 8 days of administration, the cell proliferation rate of the low concentration extract group of B material was the highest, which was significantly higher than that of the other concentration groups. The cell proliferation rate of each concentration group of A, B, C, and D materials was close to 100%, and the cell proliferation rate of more than half of the groups was ∼200%. The results of this comprehensive analysis showed that the extracts of various concentrations in each group had no significant effect on cell growth and that the cell proliferation rate showed a gradual increased trend with time.

#### 3.4.2 Cell Adhesion

**Figure 12:**
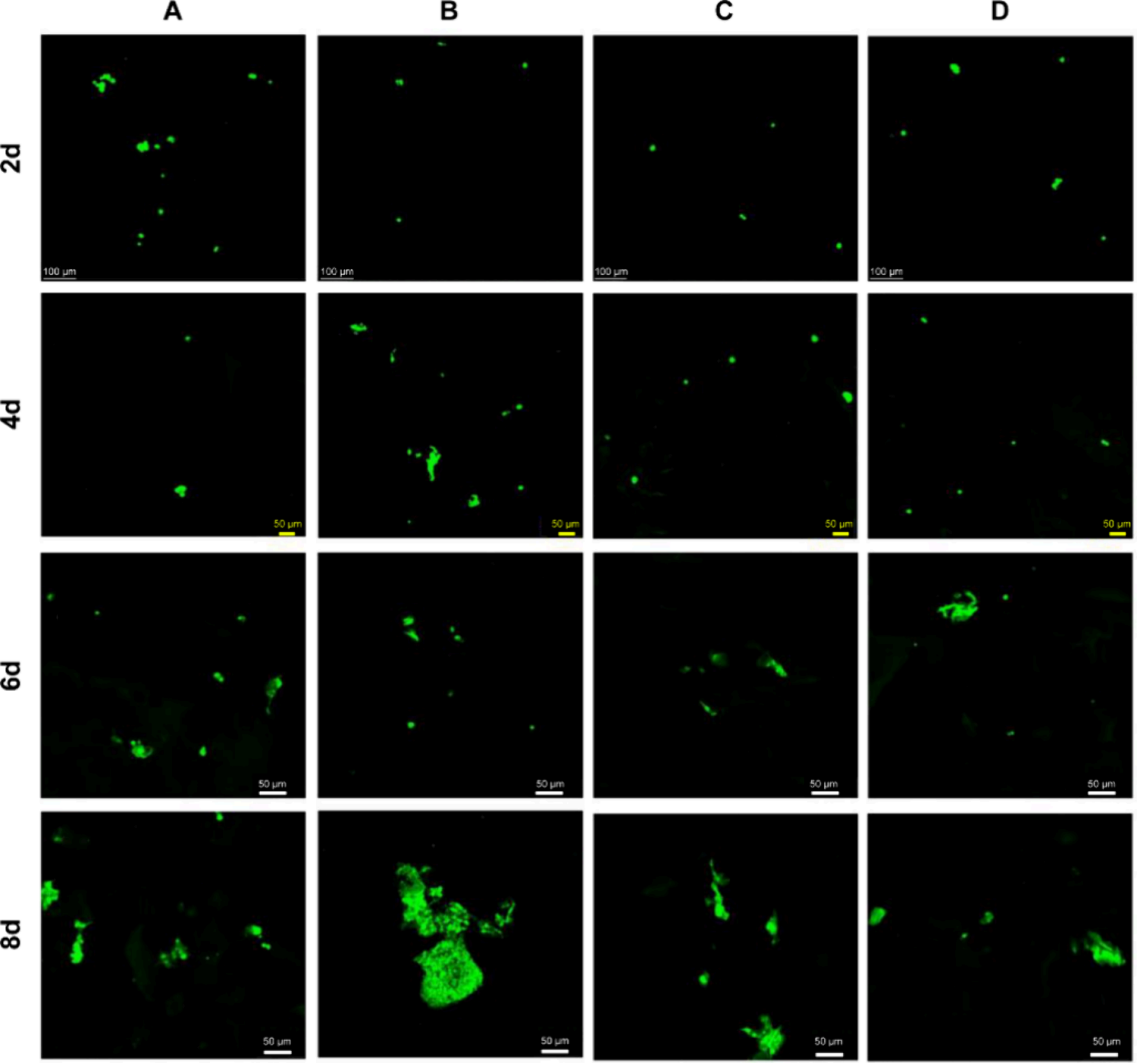
3D-reconstructed confocal images of different scaffolds (A: <0.1 μm yak skin gelatin/PMVE-MA composite scaffold materials; B: 0.1–0.22 μm yak skin gelatin/PMVE-MA composite scaffold materials; C: >0.22 μm yak skin gelatin/PMVE-MA composite scaffold materials; and D: yak skin gelatin/PMVE-MA composite scaffold materials; 2, 4, 6, and 8 days indicate the time when cells were cocultured with scaffold materials, respectively).

Figure 12 shows the growth and adhesion of HepG2 cells with GFP on the gelatin/PMVE-MA composite scaffold at 2, 4, 6, and 8 days. The cells were seeded in composite material for 2 and 4 days, with sporadic cell growth. After 6 days, cells showed aggregate growth; after day 8, cell growth was in large aggregate. Cell adhesion on group B was significantly higher than the other three groups, indicating that group B was the most suitable scaffold for cell growth, which is consistent with the results of roughness because larger roughness will create a larger area for cell adhesion.

#### 3.4.3 Cell Infiltration

Table 5 shows the growth of HepG2 cells with GFP on gelatin/PMVE-MA composite scaffolds of different molecular weights. The proliferation of cells on the composite was observed for 2, 4, 6, and 8 days. The results showed that when the cells were seeded on the composite material for 2 and 4 days, cells grew sporadically. After 6 days of seeding, cells showed aggregate growth. On the eighth day, cells gathered and grew in a large area, with the growth of cells on B group material improved. This proliferation was deemed the best observation growth for all groups, with the infiltration depth reaching 78 μm. Based on this observation, material B is the most suitable for cell growth.

**Table 5:** Depth (μm) to Which Cells Had Infiltrated in Scaffolds^a^

**scaffolds/time**	**infiltration depth (μm)**			
	**2 d**	**4 d**	**6 d**	**8 d**
A	16	18	36	96
B	12	58	60	78
C	12	16	46	48
D	0	10	34	46

a A: <0.1 μm yak skin gelatin/PMVE-MA composite scaffold materials; B: 0.1–0.22 μm yak skin gelatin/PMVE-MA composite scaffold materials; C: >0.22 μm yak skin gelatin/PMVE-MA composite scaffold materials; and D: yak skin gelatin/PMVE-MA composite scaffold materials.

## 4 Discussion

The numerous beneficial physical and chemical properties of gelatin are the basis for its wide application. Results from AFM observation of the structural changes of different molecular weight segments of gelatin show that there are certain differences in the structures of each molecular weight segment of gelatin. Among these differences, the roughness and average height of the gelatin in component B are the highest, while gelatin in component A has the most uniform particles as well as the lowest roughness. Further, the average height of gelatin B is significantly higher than the other molecular weight ranges of gelatin. This is consistent with its particularity in blood enrichment and platelet activation^23^.

Gelatin contains a large amount of glycine and proline, which may be due to the repeated Gly-Pro-Hyp sequence within gelatin and collagen molecules and the simple structure of glycine, which is the amino acid with the smallest molecular weight among the essential amino acids. The amino acid composition of yak skin gelatin is different from that of other animal gelatins that have been reported to date. For example, fish scale gelatin only possesses 15 amino acids, exhibiting lower contents of methionine, isoleucine, and histidine than those present in the yak skin^45^. Lysine was not detected in fish scale gelatin^52^. Studies conducted on the thermodynamic properties of gelatin have shown that the thermal stability of yak skin gelatin is higher than that of other mammals^53^.

Based on the difference between the basic properties of yak skin gelatin with different molecular weights, it is speculated that the properties of composite scaffolds prepared with different molecular weights of gelatin and PMVE-MA are also different. The yak skin gelatin/PMVE-MA composite scaffold material was used as a sample to test the properties of the scaffold material with different molecular weight yak skin gelatin.

The microstructure of groups A, C, and D was dense and less connected between laminar and pore structures. Group B had a loose laminar structure with more pore structures and interconnected pores, which was suitable for cell adhesion. The composite scaffold material prepared from C group gelatin with the largest molecular weight had the lowest swelling degree. A possible reason for this is that the C group gelatin itself had the largest molecular weight and that the sample solution was particularly viscous. The composite scaffold prepared by the MA reaction had no obvious interconnected pore structure, resulting in a low degree of swelling; further degradation rate experiments show that the composite scaffold prepared from gelatin with a larger molecular weight had a lower degradation rate. The reason for this may also be related to the dense structure of the molecular weight of gelatin itself. The stent material prepared from the materials of group B had strong mechanical properties; the compressive strength was extremely significant when compared to that of the materials of groups A, C, and D. Additionally, the difference in bending strength was extremely significant, being much higher than the materials of group A and also higher than the materials of groups C and D. There was no significant difference in flexural strength between groups C and D, suggesting that there was no significant correlation between the mechanical properties of scaffolds and the molecular weight of gelatin.

Biocompatibility includes hemocompatibility and cytocompatibility and mainly follows the interaction between scaffold materials and blood and scaffold materials and cells. Studies on these interactions include the effect of scaffold materials on hemolysis rate and platelet adhesion, the toxicity of scaffold materials to cells, and the effect of the materials on cell adhesion and cell infiltration depth. When considering scaffold materials for such studies, eligible requirements include antiplatelet thrombosis, antihemolysis, cell adhesion, noninhibitory cell growth, and promotion of cell adhesion and infiltration. The biocompatibility experiments of gelatin/PMVE-MA composite scaffolds with different molecular weight segments in this study show that the hemolysis rate of the materials increases with the increase of the molecular weight of gelatin, but the highest value of the hemolysis rate among the four groups of materials was still less than 1%, which is far lower than the internationally recognized 5%—ultimately meeting the hemolytic requirement of the composite scaffold material into the body. The platelet adhesion rate of the composite scaffold material is inversely proportional to the molecular weight of gelatin, and the larger the molecular weight, the less platelet adhesion.

The four groups of materials had no obvious toxicity to cells, and the cell proliferation rate gradually increased with the extension of culture time, indicating that the extracts of each group of materials had no significant effect on cell proliferation; that is, the composite materials had no obvious toxicity to cells. The surface of the scaffold material was inoculated with cells, with the first biological response of cells to the scaffold material being cell adhesion. The degree of cell adhesion is highly dependent on the surface morphology and chemical properties of the scaffold material^54^. Further affecting processes such as cell proliferation and differentiation^55^, the rough material surface helps to promote the interaction between cells and the scaffold material, thereby promoting the attached growth of cells^56^. Combined with the microstructure analysis of each group of materials, the surface pores of group B materials are interconnected, meaning that they are more suitable for cell migration. The infiltration of cells on the materials in group A was the most ideal. After 8 days of coculture, a small number of cells infiltrated at a depth of 96 μm. A possible reason for this is that the materials in group A have a lamellar structure similar to that in group B, which is convenient for cell migration and infiltration. However, the physical properties of the materials in group A are not ideal and should not be used in practice. After 8 days of coculture, the infiltration depth of group B reached 78 μm, and the number of cells reaching this depth grew, indicating that the materials in group B are more suitable for cell growth. Further, there are many pores in the lamellar structure of the group B material, and the pores are interconnected. Due to these physical properties, it has been deemed to be the most suitable choice among the four groups of materials. Based on these findings, this material should be studied further to better understand its potential applications for it.

## 5 Conclusions

In this article, yak skin gelatin was extracted and prepared, with gelatin samples of molecular weight segments separated according to the pore size of the ultrafiltration membrane. The physicochemical properties of each molecular weight segment were then studied to better understand their properties for potential applications in humans. Differences in the basic properties of each molecular weight segment and the effects of different molecular weight segments of gelatin on the composite scaffolds were included in the study. The results show that the microstructure, swelling degree, and mechanical properties of 0.1–0.22 μm yak skin gelatin are promising, with a low hemolysis rate. This component of gelatin has no obvious effect on cell proliferation, and cells grow well on this gelatin/PMVE-MA composite scaffold. This study provides a foundation for the application of yak skin in tissue engineering and shows that it has great potential in tissue engineering.

## Data Availability

The raw processed data required to reproduce these findings cannot be shared at this time as the data also form part of an ongoing study.
